# *Notes from the Field:* Trends in Emergency Department Visits for Firearm Injuries — United States, January 2018–December 2023

**DOI:** 10.15585/mmwr.mm7346a4

**Published:** 2024-11-21

**Authors:** Kristin M. Holland, Yushiuan Chen, Marissa L. Zwald, Steven A. Sumner, Katherine A. Fowler, Michael Sheppard, Thomas R. Simon

**Affiliations:** ^1^Division of Violence Prevention, National Center for Injury Prevention and Control, CDC; ^2^Office of Public Health Data, Surveillance, and Technology, CDC.

**Summary**

**What is already known about this topic?**

Fatal and nonfatal firearm injuries increased in 2020, coinciding with the COVID-19 pandemic and other sociocultural factors, and remained significantly higher during 2021–2022 than before the onset of the pandemic.

**What is added by this report?**

CDC’s National Syndromic Surveillance Program data show that although firearm injury–related emergency department visit rates increased significantly during 2018–2020 and then decreased during 2021–2023, rates remain higher than those in 2019.

**What are the implications for public health practice?**

Firearm injury prevention warrants a comprehensive public health approach. CDC’s suite of Prevention Resources for Action outlines the best available evidence for preventing injuries, including firearm injuries, related to suicide and community, intimate partner, and other forms of violence.

Research has highlighted recent increases in fatal and nonfatal firearm injuries (*1*–*3*). For example, from 2019 to 2020, firearm homicides and nonfatal firearm injury–related emergency department (ED) visits increased approximately 35% and 37%, respectively (*1*,*2*). These increases coincided in part with the COVID-19 pandemic and other sociocultural factors, such as strained law enforcement–community relationships, increases in firearm purchases and some types of crime, and increased discussion about systemic inequities (e.g., in economic, educational, and employment opportunities) that were potentially exacerbated by pandemic conditions (*1*–*5*). A year-over-year comparison of firearm injury–related ED visits could provide important insight into the direction of trends, which can help guide ongoing firearm injury prevention efforts.

## Investigation and Outcomes

CDC’s National Syndromic Surveillance Program (NSSP) data from consistently reporting EDs* were used to assess mean monthly counts of firearm injury–related ED visits by year during 2018–2023. Year-over-year comparisons (i.e., comparison of a metric from 1 year to the same period in the previous year) of visit ratios (VRs) of firearm injury–related ED visit rates with 95% CIs were examined overall and by sex and age group; 95% CIs that exclude 1 were considered statistically significant.^†^ Injury intents identified by the firearm injury–related ED visit syndrome definition include unintentional, intentional self-directed, assault, undetermined intent, legal intervention, and terrorism. This activity was reviewed by CDC, deemed not research, and was conducted consistent with applicable federal law and CDC policy.^§^

NSSP data included approximately 425 million ED visits during 2018–2023, including 338,390 that involved a firearm injury, for an average annual rate of 81.2 per 100,000 ED visits (CDC, NSSP, unpublished data, 2024). Mean monthly counts of firearm injury–related ED visits ranged from 3,754.4 in 2018 to 5,559.0 in 2020 (Table). Firearm injury–related ED visit rates increased significantly from 2018 to 2019 (VR = 1.04) and again from 2019 to 2020 (VR = 1.75). Since their peak in 2020 (mean monthly rate = 114.7 per 100,000 ED visits; CDC, NSSP, unpublished data, 2024), firearm injury–related ED visit rates decreased significantly year over year (VR from 2020 to 2021_,_ from 2021 to 2022, and from 2022 to 2023 were 0.82, 0.87, and 0.92, respectively) but have not returned to the level of the 2019 mean monthly rate (64.3 per 100,000 ED visits; CDC, NSSP, unpublished data, 2024). Patterns of firearm injury–related ED visit rates by sex mirrored those for firearm injury ED visits overall and were also reflected in monthly visit counts. Specifically, visit rates among both females and males increased during 2018–2020 and decreased yearly thereafter, with the most pronounced decreases observed during 2021–2022 among males aged ≥65 and 25–34 years (34.3% and 14.6% decreases, respectively) and among females aged ≥65 and 0–14 years (24.0% and 14.1% decreases, respectively). Overall firearm injury–related ED visit counts in 2023 were 13.5% higher than in 2019, and VRs were significantly higher for females and males of all ages, except those aged ≥65 years.

**TABLE:** Mean monthly number of emergency department visits, percent change* in emergency department visits, and visit ratios^†^ of emergency department visits for firearm injury,^§^ overall and by sex and age group — National Syndromic Surveillance Program,^¶^ United States, January 2018–December 2023

Sex/Age group, yrs	2018	2019		2020		2021		2022		2023		
	Mean firearm injury ED visits per mo	Mean firearm injury ED visits per mo	% Change 2018–2019	Mean firearm injury ED visits per mo	% Change 2019–2020	Mean firearm injury ED visits per mo	% Change 2020–2021	Mean firearm injury ED visits per mo	% Change 2021–2022	Mean firearm injury ED visits per mo	% Change 2022–2023	% Change 2019–2023
**All**	**3,754.40**	**4,050.20**	**7.9**	**5,559.00**	**37.3**	**5,357.70**	**−3.6**	**4,881.40**	**−8.9**	**4,596.40**	**−5.8**	**13.5**
** VR (95% CI)**	**—**	**—**	1.04 (1.03–1.05)**	**—**	**1.75 (1.73–1.77)^††^**	**—**	**0.82 (0.81–0.83)^§§^**	**—**	**0.87 (0.86–0.88)^¶¶^**	**—**	0.92 (0.90–0.93)***	**1.14 (1.13–1.15)^††^**
**Female**												
Overall	498.2	566.1	13.6	784.7	38.6	787.2	0.3	725.4	−7.9	685.1	−5.6	21.0
VR (95% CI)	—	—	1.11 (1.07–1.15)**	—	1.81 (1.76–1.87)^††^	—	0.85 (0.82–0.87)^§§^	—	0.87 (0.84–0.89)^¶¶^	—	0.91 (0.88–0.94)***	1.21 (1.18–1.25)^††^
0–14	26.4	26.9	1.9	45.0	67.2	42.7	−5.1	36.7	−14.1	36.3	−1.1	34.9
VR (95% CI)	—	—	0.97 (0.83–1.13)**	—	3.21 (2.80–3.69)^††^	—	0.64 (0.56–0.72)^§§^	—	0.70 (0.61–0.79)^¶¶^	—	1.02 (0.89–1.16)***	1.45 (1.25–1.67)^††^
15–24	151.2	183.4	21.3	258.1	40.7	254.3	−1.5	236.1	−7.2	211.8	−10.3	15.5
VR (95% CI)	—	—	1.20 (1.13–1.27)**	—	1.86 (1.76–1.96)^††^	—	0.84 (0.79–0.88)^§§^	—	0.91 (0.87–0.96)^¶¶^	—	0.89 (0.85–0.94)***	1.26 (1.19–1.34)^††^
25–34	132.1	147.8	11.9	219.4	48.4	211.5	−3.6	193.6	−8.5	177.0	−8.6	19.8
VR (95% CI)	—	—	1.10 (1.03–1.18)**	—	1.87 (1.76–1.99)^††^	—	0.85 (0.80–0.89)^§§^	—	0.91 (0.86–0.96)^¶¶^	—	0.89 (0.84–0.94)***	1.28 (1.20–1.36)^††^
35–64	142.7	164.0	14.9	201.2	22.7	215.1	6.9	194.1	−9.8	198.9	2.5	21.3
VR (95% CI)	—	—	1.13 (1.06–1.20)**	—	1.51 (1.42–1.60)^††^	—	0.94 (0.89–0.99)^§§^	—	0.88 (0.83–0.93)^¶¶^	—	0.98 (0.92–1.04)***	1.22 (1.15–1.29)^††^
≥65	30.8	29.8	−3.2	39.4	32.5	40.4	2.5	30.7	−24.0	26.8	−12.7	−10.1
VR (95% CI)	—	—	0.92 (0.79–1.06)**	—	1.62 (1.41–1.86)^††^	—	0.88 (0.77–1.00)^§§^	—	0.69 (0.60–0.78)^¶¶^	—	0.82 (0.70–0.95)***	0.80 (0.68–0.92)^††^
**Male**												
Overall	3,201.1	3,436.1	7.3	4,701.5	36.8	4,493.2	−4.4	4,065.8	−9.5	3,827.2	−5.9	11.4
VR (95% CI)	—	—	1.04 (1.02–1.05)**	—	1.70 (1.67–1.72)^††^	—	0.82 (0.81–0.83)^§§^	—	0.86 (0.85–0.87)^¶¶^	—	0.92 (0.90–0.93)***	1.09 (1.08–1.11)^††^
0–14	73.7	87.3	18.5	116.3	33.2	120.2	3.4	118.5	−1.4	119.3	0.7	36.7
VR (95% CI)	—	—	1.13 (1.03–1.23)**	—	2.60 (2.40–2.82)^††^	—	0.68 (0.63–0.73)^§§^	—	0.79 (0.73–0.85)^¶¶^	—	1.03 (0.96–1.11)***	1.45 (1.34–1.57)^††^
15–24	1,094.2	1,170.8	7.0	1,601.8	36.8	1,469.8	−8.2	1,330.8	−9.5	1,256.4	−5.6	7.3
VR (95% CI)	—	—	1.04 (1.02–1.07)**	—	1.73 (1.70–1.77)^††^	—	0.79 (0.77–0.81)^§§^	—	0.88 (0.86–0.90)^¶¶^	—	0.94 (0.91–0.96)***	1.13 (1.10–1.16)^††^
25–34	928.6	996.5	7.3	1,398.7	40.4	1,303.5	−6.8	1,112.6	−14.6	1,007.9	−9.4	1.1
VR (95% CI)	—	—	1.05 (1.02–1.07)**	—	1.61 (1.57–1.64)^††^	—	0.85 (0.83–0.87)^§§^	—	0.88 (0.86–0.90)^¶¶^	—	0.89 (0.87–0.91)***	1.07 (1.04–1.10)^††^
35–64	814.7	884.8	8.6	1,190.2	34.5	1,209.2	1.6	1,122.5	−7.2	1,077.8	−4.0	21.8
VR (95% CI)	—	—	1.06 (1.03–1.09)**	—	1.52 (1.48–1.56)^††^	—	0.92 (0.90–0.94)^§§^	—	0.93 (0.91–0.95)^¶¶^	—	0.93 (0.91–0.95)***	1.20 (1.17–1.24)^††^
≥65	192.2	207.8	8.1	272.3	31.0	251.5	−7.6	165.3	−34.3	169.8	2.7	−18.3
VR (95% CI)	—	—	1.02 (0.96–1.08)**	—	1.47 (1.40–1.55)^††^	—	0.82 (0.78–0.86)^§§^	—	0.60 (0.57–0.64)^¶¶^	—	0.96 (0.90–1.02)***	0.70 (0.66–0.74)^††^

**Abbreviations**: ED = emergency department; NSSP = National Syndromic Surveillance Program; VR = visit ratio.

* Percent change in visits per week during each surveillance period was calculated as ([mean monthly ED visits for firearm injury during surveillance period − mean monthly ED visits for firearm injury during comparison period] / mean monthly ED visits for firearm injury during comparison period) × 100.

^†^ VR = (ED visits for firearm injury during surveillance period / all ED visits during surveillance period) / (ED visits for firearm injury during comparison period / all ED visits during comparison period). Ratios >1 indicate a higher proportion of ED visits for firearm injury during the surveillance period than the comparison period; ratios <1 indicate a lower proportion during the comparison period than during the surveillance period; 95% CIs that do not include 1 were considered statistically significant.

^§^ ED visits for an initial firearm injury encounter were identified by querying a categorization developed and validated by CDC in partnership with state, tribal, local, and territorial health departments. The following intent types were included in the definition: unintentional, intentional self-directed, assault, undetermined intent, legal intervention, and terrorism.

^¶^ NSSP is a collaboration among CDC, local and state health departments, and federal, academic, and private sector partners. NSSP receives medical record data from approximately 80% of EDs nationwide, although fewer than 50% of facilities from California, Hawaii, Minnesota, and Oklahoma currently participate in NSSP. To reduce impact of reporting pattern changes, which can vary across jurisdictions, analyses were restricted to facilities with a coefficient of variation of <40% for total visit volume and with >75% complete information on discharge diagnoses during 2018–2023: the final sample represents 1,794 facilities (43% of total facilities that contribute data to NSSP). https://www.cdc.gov/nssp/index.html

** Comparison period is calendar year 2018 (January–December 2018).

^††^ Comparison period is calendar year 2019 (January–December 2019).

^§§^ Comparison period is calendar year 2020 (January–December 2020).

^¶¶^ Comparison period is calendar year 2021 (January–December 2021).

*** Comparison period is calendar year 2022 (January–December 2022).

## Preliminary Conclusions and Actions

Approximately 50,000 firearm-related deaths and twice as many firearm injuries than deaths occur in the United States every year.^¶^ Although firearm injury–related ED visit rates decreased during 2021–2023, they remain high. The prevalences of firearm injury and associated death suggest the need for continued monitoring and a comprehensive prevention approach. National, state, and local data available through CDC’s NSSP and the Firearm Injury Surveillance Through Emergency Rooms (FASTER) program** can be used as a tool for planning and evaluating prevention efforts. In addition, CDC’s suite of Prevention Resources for Action^††^ describes the best available evidence for preventing suicide and community, intimate partner, and other forms of violence, including firearm-related injuries. Highlighted prevention programs, practices, and policies include teaching coping, problem-solving, and healthy relationship skills; street outreach and hospital-based violence prevention programs; place-based prevention, including cleaning vacant lots and secure firearm storage; and strengthening household financial security through economic supports. States and communities can use these resources to guide local firearm violence and suicide prevention efforts.

## Footnotes

* Electronic health record data from EDs participating in CDC’s NSSP (https://www.cdc.gov/nssp/index.html), which covers approximately 80% of U.S. EDs, are included in this report. To reduce impact of reporting pattern changes, which vary across jurisdictions, analyses were restricted to facilities with a coefficient of variation of <40% for total visit volume and with >75% complete information on discharge diagnoses during 2018–2023; the final sample represents 1,794 facilities (43% of total facilities that contribute data to NSSP).

† ED visit counts for initial firearm injury encounters were identified using the CDC Firearm Injury v2 syndrome definition (https://knowledgerepository.syndromicsurveillance.org/cdc-firearm-injury-v2). VRs of firearm injury rates were calculated as (ED visits for firearm injury during surveillance period / all ED visits during surveillance period) / (ED visits for firearm injury during comparison period / all ED visits during comparison period). Ratios >1 indicate a higher proportion of ED visits for firearm injury during surveillance period than during the comparison period.

§ 45 C.F.R. part 46, 21 C.F.R. part 56; 42 U.S.C. Sect. 241(d); 5 U.S.C. Sect. 552a; 44 U.S.C. Sect. 3501 et seq.

¶
https://wisqars.cdc.gov/index.html

** CDC’s FASTER program encompasses two initiatives funded in 2020 and 2023 to collect, analyze, and disseminate data on violence-related ED visits, including firearm injuries, in near real time. https://www.cdc.gov/firearm-violence/php/funded-surveillance/index.html

††
https://www.cdc.gov/violence-prevention/php/resources-for-action/index.html
